# Retraction: Tregs depletion aggravates activation of astrocytes by modulating IL-10/GXP4 following cerebral infarction

**DOI:** 10.3389/fimmu.2024.1513350

**Published:** 2024-10-25

**Authors:** Frontiers Editorial Office

The journal retracts the 2023 article cited above.

Following publication, concerns were raised regarding inaccuracies in several sections of the article. The raw data and materials provided by the authors were incomplete and contained errors, not allowing for the results to be reproduced. Further assessment by the Chief Editor led to the conclusion that the article would require an extensive number of corrections that would substantially modify the content from the version assessed during peer review, as the article’s conclusions and assertions are not sufficiently supported by the findings from the material provided. Therefore, the article has been retracted.

This retraction was approved by the Chief Editor of Frontiers in Immunology and the Chief Executive Editor of Frontiers.

